# Experiments on the Absorbent Power of the Bladder

**Published:** 1870-09

**Authors:** 


### Experiments on the Absorbent Power of the Bladder.

Physicians and physiologists are by no means agreed on the power of absorption possessed by the mucous membrane of the urinary bladder. While some assert that the excrementitious substances of the urine would constantly be carried back to the circulation if this absorption took place, others deny this fact. M. Beclard asserts that the fluids contained in the excretory reservoirs (the urinary and biliary bladders) are in the process of absorption. We should remember that the epithelial lining membrane of these viscera consists of stratified layers of pavement epithelium, which is less easily traversed by fluids than the simple membrane of the serous cavities, Nevertheless a slight absorption takes place in these organs. The morning urine, which has remained a part of the night in the bladder, is of a darker color than the urine of the day, and also than that which has been passed after taking fluids.

Recent experiments of Bert and Joylet confirm those of M. Ségalas, and demonstrate the fact that the bladder, in health, rapidly absorbs certain substances introduced into it. A solution of strychnia, one part in one hundred, was carefully injected into the bladders of several rabbits. causing their death at the end of four minutes, although a few milligrammes only of the salt had been introduced, and a part of this had been removed by injections of water on the first atpearance of symptoms of poisoning. Autopsy showed that the mucous membrane of the viscus remained healthy. Injections of a solution of atropia did not kill the animal, or sensibly diminish the pupil.

The possibility of causing certain substances to be absorbed by the healthy bladder offers a therapeutic resource of considerable importance; in cholera, for instance, where medicine cannot be absorbed by the stomach and intestines, advantage can be taken of the circumstance that the bladder is habitually empty, for the injection of certain solutions which experience has shown to be absorbed. During an epidemic of cholera, M. Brown Sequard injected into the bladder of several patients laudanum and alkaline carbonates, and he was convinced that these substances were absorbed in considerable amounts. Experiments of a similar character have been tried in Germany and Italy.—*L' Union Med. Med. and Surg. Journ.*

